# Hypofunction of directed brain network within alpha frequency band in depressive patients: a graph-theoretic analysis

**DOI:** 10.1007/s11571-022-09782-6

**Published:** 2022-02-05

**Authors:** Shuang Liu, Sitong Chen, Zhenni Huang, Xiaoya Liu, Meijuan Li, Fangyue Su, Xinyu Hao, Dong Ming

**Affiliations:** 1 School of Precision Instruments and Optoelectronics Engineering, Tianjin University, Tianjin, China; 2 Academy of Medical Engineering and Translational Medicine, Tianjin University, Tianjin, China; 3 Anding Hospital, Tianjin, China

**Keywords:** Depression, Alpha frequency band, Partial directed coherence, Weighted directed brain network, Disruption of directed connectivity, Correlation

## Abstract

Directed brain networks may provide new insights into exploring physiological mechanism and neuromarkers for depression. This study aims to investigate the abnormalities of directed brain networks in depressive patients. We constructed the directed brain network based on resting electroencephalogram for 19 depressive patients and 20 healthy controls with eyes closed and eyes open. The weighted directed brain connectivity was measured by partial directed coherence for α, β, γ frequency band. Furthermore, topological parameters (clustering coefficient, characteristic path length, and et al.) were computed based on graph theory. The correlation between network metrics and clinical symptom was also examined. Depressive patients had a significantly weaker value of partial directed coherence at alpha frequency band in eyes-closed state. Clustering coefficient and characteristic path length were significantly lower in depressive patients (both *p* < .01). More importantly, in depressive patients, disruption of directed connectivity was noted in left-to-left (*p* < .05), right-to-left (*p* < .01) hemispheres and frontal-to-central (*p* < .01), parietal-to-central (*p* < .05), occipital-to-central (*p* < .05) regions. Furthermore, connectivity in LL and RL hemispheres was negatively correlated with depression scale scores (both *p* < .05). Depressive patients showed a more randomized network structure, disturbed directed interaction of left-to-left, right-to-left hemispheric information and between different cerebral regions. Specifically, left-to-left, right-to-left hemispheric connectivity was negatively correlated with the severity of depression. Our analysis may serve as a potential neuromarker of depression.

## Introduction

Characterized by high level of anhedonia and continuous pessimism (DeRubeis et al. 2008; Kessler et al. 2003), depression is one of the most serious and also common mental disease. According to the World Health Organization, there are about 350 million persons in the world suffering from depressive disorders. However, the pathological mechanism of depression is still not clear, and the methods of clinical diagnosis of depression are somewhat subjective (Hulshoff Pol and Bullmore 2013), which has brought serious psychological and economic burden to people. It is imperative to explore the pathological functional mechanism of this mental disorder to improve its detection and treatment efficiency. With the development of brain imaging technology, researchers have found that human brain is one of the most complex systems in nature, in which a large number of neurons form a highly complex network to enable information processing and cognitive expression (Bassett and Bullmore 2006). Brain networks can contribute to understanding the structure, function, and pathological mechanisms of the brain, which can help prevent, diagnose, and treat mental diseases.

Findings with a specific focus on brain networks have revealed that the structural and functional networks of human brain conform to a complex network model (Bullmore and Sporns 2009; van den Heuvel and Hulshoff Pol 2010). Graph theory provides a promising tool to explore computational models of brain networks and quantitatively describes the connectivity of different brain regions (Kaiser 2011; Mikail Rubinov and Sporns 2010). As a common analytical approach for complex systems, such as human brain, graph theory has indeed been applied to explore the functional and structural abnormalities in mental disorders, such as depression, Alzheimer’s disease, schizophrenia, and bipolar disorder (Leistedt et al. 2009; Micheloyannis et al. 2006; Rubinov et al. 2009; Stam et al. 2006, 2008; Strakowski et al. 2012). The topological network parameters are typically evaluated to quantify the properties of these mental disorders, which then provide the diagnostic criteria at the global and local levels. Recently, alterations in the structure and functions of brain networks have been frequently discovered in depressive patients (DP) using graph theory (Stam and Reijneveld 2007). For example, an electroencephalogram (EEG)-based study revealed that DP showed significant randomization of global network metrics; they were characterized by lower characteristic path length at the delta and theta bands compared with healthy controls (HC) (Leistedt et al. 2009). A study based on resting state functional magnetic resonance imaging (fMRI) data from DP also found a decrease in characteristic path length, an increase in global efficiency, and changes in other metrics, such as reduced clustering coefficient and local efficiency (Li et al. 2017). In another study, researchers constructed the brain functional network by collecting task-state fMRI data of 13 major depressive disorder (MDD) patients and found that the global efficiency of the brain functional network decreased and the local efficiency increased when MDD patients performed negative and neutral emotion processing tasks (Park et al. 2014). The decreased global efficiency of the dorsal striatum, inferior frontal gyrus, orbitofrontal cortex, occipital lobe, and somatosensory cortex in MDD patients was also shown in an fMRI-based study (Meng et al. 2014).

However, conventional structural or functional network analysis considers only the undirected information connectivity, whereas the transmission of information in the brain appears to be directed. For example, a preferential direction of information flow was found in the delta and beta rhythms across wake to sleep states: preferred right-to-left hemisphere direction for delta and left-to-right for beta rhythms (Bertini et al. 2007). Consequently, studies about information flow, especially the directed connectivity between different cerebral hemispheres as well as cerebral regions, may contribute to uncovering more information about the functions and the structure of the brain. Previous studies have suggested that the interactions between the left and right cerebral hemispheres play a crucial role in cognitive and emotional processing, which contributes to mediating the symptoms of depression (Banich et al. 1992; Compton et al. 2005; Toro et al. 2008). Besides, it is also necessary to investigate the functional coordination of different cerebral regions. For instance, the frontoparietal control systems contribute to promoting and maintaining mental health (Cole et al. 2014). Directed analysis may have considerable impact on the studies for dysfunctions of DP. To our knowledge, few studies have yielded some results on depression through directed network research. The directed functional connectivity between cerebral hemispheres and between different cerebral regions is still not clearly known. To generalize the study of directed functional connectivity, effective connectivity, which deals with causal interactions of brain regions, can be used to address this obstacle. Partial directed coherence (PDC) is one of the main methods used to quantify the effective connectivity between different channels. It neglects the cross-channel directed interdependence that can be extracted from multivariate data. This cortical interdependence is essential to understanding inter- and intra-hemispheric as well as different regions’ causal interactions (Sun et al. 2008). Indeed, PDC has been used to simulate a neural network to help further research about the functions of brain because of its ability to reveal the direction of information flow (Sameshima and Baccalá, 1999). This is a promising tool to study the aberration of directed functional connectivity in various brain disorders (Coito et al. 2016; Ana Coito et al. 2019; Sperdin et al. 2018).

To investigate alterations of the directed connectivity and the topological network parameters in DP, we explored differences in the weighted directed functional brain network between DP (N = 19) and HC (N = 20) in resting state with eyes closed (EC) and eyes open (EO). We first examined the differences of the mean value of partial directed coherence over all paired channels between the two groups. Then, graph theory was applied to construct the weighted directed network for subsequent analysis. Conventional topological parameters including the clustering coefficient, characteristic path length and global efficiency were computed. What’s more, we presented the inter- and intra-hemispheric connectivity matrices and statistical significance of different groups as well as the significant differences of directed connectivity between cerebral regions. In addition, the differences in the local characteristics between the two groups were compared, namely, local efficiency and out- and in-strength. Furthermore, the Pearson correlation coefficients between depression symptom measures and network metrics were computed. We hypothesized abnormalities in topological parameters and a disrupted connectivity of different cerebral hemispheres and regions in DP compared with HC.

## Methods

### Subjects

Depressive patients (N = 19) and healthy controls (N = 20) with no prior or current history of depression were recruited to participate. All of them were right-handed. The age of depressive patients (female/male = 11/8) ranged from 21 to 31 years (mean = 26.53; standard deviation (SD) = 3.49), and the age of healthy controls (female/male = 11/9) ranged from 21 to 31 (mean = 25.00; standard deviation = 2.29). There were no significant between-group differences in age (${\text{t}} = - 1.6233, p = .1130$) or gender (${\upchi }^{2} = .0332,{ }p{ } = { }.8554$).

Depressive patients from Tianjin Anding Hospital, Tianjin, China and The Second Xiangya Hospital, Changsha, China were diagnosed and recommended by two clinical psychiatrists. The healthy controls were all recruited from Tianjin University. The study was approved by the Ethics Committee of Tianjin Anding Hospital and the Ethics Committee of The Second Xiangya Hospital. All patients received a Structured Clinical Interview for Diagnostic and Statistical Manual of Mental Disorders (DSM)-IV. The inclusion criteria for depressive patients were consistent with DSM-IV criteria: Hamilton Depression Rating Scale (HDRS) scores of 17 or greater on the 17-item scale. All the patients were first-episode untreated.

Exclusion criteria were the following: previous history of psychiatric genetic disorders or other psychiatric diseases, severe physical diseases, other medical drugs before the trial, intellectual or behavioral disorders, a history of alcohol and drug abuse, and participating in clinical trials of other drugs.

### Experiment

This study was conducted on the basis of an 8 min resting state experiment that was performed in a quiet environment without exogenous interference. The procedure of the study was clearly explained to all subjects. During the experiment, each subject was prompted to remain quiet and relaxed with their eyes open and eyes closed in two alternating orders by voice playback.

### EEG recordings and preprocessing

Left ear mastoid process (M1) was selected as the reference electrode, and the EEG data of 30 conductive electrodes were finally recorded using the Neuroscan SYNAMPS based on the international 10–20 system (30 channels = FP1, FP2, FZ, F4, F8, F3, F7, FCZ, FC4, FT8, FC3, FT7, CZ, C4, T8, T7, C3, TP7, TP8, P7, CPZ, CP4, CP3, PZ, P4, P8, P3, OZ, O2, and O1). Sampling frequency was 1000 Hz, and the channel impedances were maintained less than 10 kΩ.

EEG preprocessing was performed using EEGLAB in MATLAB (R2016a), which is an open-source MATLAB-based toolbox for data processing of EEG signals. First, the data were re-referenced against the binaural mean reference (M1 and M2). The signals were band-pass filtered within 0.1–100 Hz and then down-sampled to 500 Hz. The 8 min signals were extracted into two sections: 4 min eyes open and 4 min eyes closed. Finally, independent component analysis (ICA) was used to remove signal artifacts caused by eye movements and breathing. We used the ADJUST1.1 toolbox in EEGLAB to help remove the artifacts, which can help users automatically filter out unnecessary ICA components and reduce errors caused by insufficient prior knowledge (Mognon et al. 2011). Frequency bands of interest were classified by alpha (8–13 Hz), beta (13–30 Hz) and gamma (30–50 Hz) respectively.

### Partial directed coherence for EEG connectivity

PDC was computed between all pairs of EEG channels at each frequency band as a measure of directed functional connectivity. PDC is a frequency-domain approach to describe directed interactions among multivariate time series. It is a normalized index showing the degree of directional linear interdependence between pairs of variables at each frequency (Sameshima and Baccalá 1999). In a linear framework, the notion of Granger-causality is closely related to vector autoregressions. The mathematical details of PDC can be briefly described as follows.

EEG is taken as an example to explain. Assume that the original EEG is a matrix of K channels:

$$Y\left( n \right) = \left[ {y_{1} \left( n \right), \ldots ,y_{K} \left( n \right)} \right]^{T}$$

$y_{{\text{i}}} \left( n \right)$ represents the EEG signal in channel *i*. Then, a vector autoregressive (van den Heuvel and Hulshoff Pol) model of order *p* for $Y\left( n \right)$ is defined as

$$Y\left( n \right) = \sum\limits_{r = 1}^{p} {A_{r} Y\left( {n - r} \right) + E\left( n \right)}$$

where ${A}_{r}$ is the calculated K * K coefficient matrix of the model using ARfit, a toolbox of Matlab (Schnieder and Neumaier 2001). *E(n)* is the error between the current value and the predicted value. Next, a representation of Granger causality in the frequency domain can be obtained from the Fourier transform of ${A}_{r}$ (Baccalá and Sameshima 2001)

$$A\left( f \right) = I - \sum\limits_{r = 1}^{p} {A_{r} e^{ - i2\pi fr} }$$

In this case, the equation denotes the difference between the n-dimensional identity matrix *I* and the Fourier transform of the coefficient series. Then the PDC value of channel *j* to channel *i* is defined as $PDC_{j \to i} \left( f \right) = {{\left| {A_{ij} \left( f \right)} \right|} \mathord{\left/ {\vphantom {{\left| {A_{ij} \left( f \right)} \right|} {\sqrt {\sum\limits_{k} {\left| {A_{kj} \left( f \right)} \right|^{2} } } }}} \right. \kern-\nulldelimiterspace} {\sqrt {\sum\limits_{k} {\left| {A_{kj} \left( f \right)} \right|^{2} } } }}$. *PDC*_*j→i*_ represents the ratio of information flowing from *j* to *i* to all information flowing from *j*. Simply put, the PDC value reflects the influence of channel *j* on channel *i*, which accounts for the proportion of its influence on other channels.

### Construction of the weighted directed brain network

We constructed the brain network using graph theory. The network was represented by a matrix with *N* nodes and *K* edges, where nodes indicated the electrodes and edges indicated the value of PDC. In this study, we got 30 electrodes. PDC was computed for every pair of electrodes for every subject in every frequency band. After calculating the value of PDC of each subject in the different groups, the PDC matrix *A*_*ij*_ (*i, j* = 1,2, …, *M*; here *M* = 30) was obtained. The diagonal of each PDC matrix was set to 0. Therefore, there were 39 matrices (30 × 30) for 39 subjects totally. The weighted directed brain network could be plotted based on these PDC matrices. The element *a*_*ij*_ in the PDC matrix indicated the weights of edges from the *j*_*th*_ electrode to *i*_*th*_ electrode. The mean directed functional brain network graph of each group was eventually obtained.

### Network analysis

Five common graph theory metrics were used to analyze the properties of the network. All of these graph theory metrics were calculated by Brain Connectivity Toolbox (Mikail Rubinov and Sporns 2010).

The weights of edges connecting to a node *i* is called the strength *W*_*i*_. The higher the strength of a node, the more important it is. In a directed graph, the strength is divided into in-strength (the weights of edges that flow from other nodes to the node) and out-degree (the weights of edges that flow out of the node).

$$W_{i} = \mathop \sum \limits_{j} w_{ij}$$

The clustering coefficient *C* is used to describe the extent of clustering between nodes in a graph. The clustering coefficient *C*_*i*_ of a node *i* is defined as the number of existing edges between neighbors of *i* divided by the maximum possible number of edges between neighbors. The average *C*_*i*_ of all nodes is *C*,

$$C = \frac{1}{n}\mathop \sum \limits_{i} \frac{1}{2}\frac{{\left[ {\left( {P^{{\left[ {{\raise0.7ex\hbox{$1$} \!\mathord{\left/ {\vphantom {1 3}}\right.\kern-\nulldelimiterspace} \!\lower0.7ex\hbox{$3$}}} \right]}} } \right) + \left( {P^{T} } \right)^{{\left[ {{\raise0.7ex\hbox{$1$} \!\mathord{\left/ {\vphantom {1 3}}\right.\kern-\nulldelimiterspace} \!\lower0.7ex\hbox{$3$}}} \right]}} } \right]_{ii}^{3} }}{{\left[ {k_{i} \left( {k_{i} - 1} \right) - 2\sum a_{ij} a_{ji} } \right]}}$$

where $$P^{{\left[ {{\raise0.7ex\hbox{$1$} \!\mathord{\left/ {\vphantom {1 3}}\right.\kern-\nulldelimiterspace} \!\lower0.7ex\hbox{$3$}}} \right]}}$$ is defined as $$\left\{ {w_{ij}^{\frac{1}{3}} } \right\}$$, i.e., the matrix obtained by taking the 3rd root of each entry (Fagiolo 2007), and $$k_{i}$$ is defined as the sum of all edges connected to node *i*.

Another parameter is the characteristic path length *L*. It is a global characteristic that indicates how easy it is to transport information in the network. The characteristic path length *L* is usually defined as the mean of the shortest path lengths between all possible pairs of nodes,

$$L = \frac{1}{n}\mathop \sum \limits_{i} \frac{{\mathop \sum \limits_{j} d_{ij}^{w} }}{n - 1}$$

where $d_{ij}^{w}$ is the shortest weighted path length (distance) between nodes *i* and *j*.

However, this original definition of *L* is problematic in networks that comprise more than one component because there exist nodal pairs that have no connecting path. A harmonic mean distance is used to measure *L*, which is called the global efficiency *G*_*e*_ (Achard and Bullmore 2007; Stam and Reijneveld 2007). *G*_*e*_ is also used to describe the global characteristics. The local efficiency *L*_*e*_, defined as the mean of the efficiencies of all subgraphs of neighbors of each of the nodes of the graph, is used to describe the local characteristics.

$$L = \frac{1}{n}\mathop \sum \limits_{i} \frac{{\mathop \sum \limits_{j} (d_{ij}^{w} )^{ - 1} }}{n - 1}$$

### Statistical analysis

The normality of data was checked using the Kolmogorov–Smirnov test. All of the data were normally distributed, thus inter-group comparisons of network metrics were conducted with t-test. Any test that yielded a *p*-value of 0.05 or less were considered statistically significant at an alpha level of 0.05. The Benjaminiand-Hochberg false discovery rate correction (BH-FDR) was performed to correct the *p*-value (Benjamini and Hochberg 1995).

## Results

### Partial directed coherence per frequency band in EO state and EC state

**Fig. 1:**
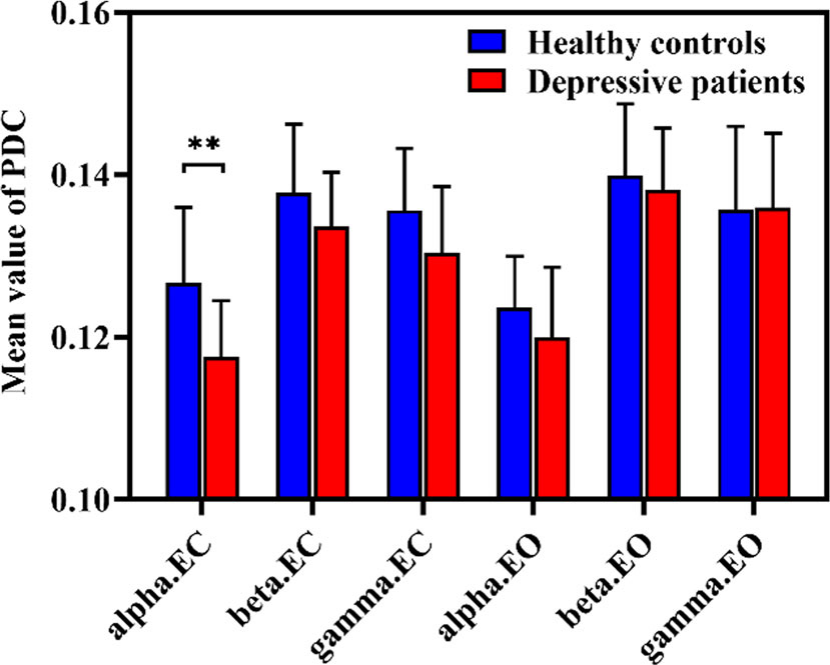
The mean value of PDC at the alpha, beta, and gamma frequency bands in EC state and EO state. The mean and standard deviation are represented by bars and lines, respectively, with blue for healthy controls and red for depressive patients. The asterisks denote corrected *p* < .01 (t-test)

Since eyes-open and eyes-closed state could exert an influence on the EEG oscillation, the mean values of PDC (averaged over all pairwise combinations of channels) at different frequency bands (alpha, beta, and gamma) were first calculated in both EO state and EC state, shown in Fig. 1. As can be seen, DP tended to show a lower value of PDC at all frequency bands in EC state, whereas there was no such trend in EO state. However, the significant difference after BH-FDR correction was only found at alpha band in EC state, where DP had a significantly lower value of PDC than HC ($0.1176{ } \pm 0.0069{\text{ versus }}0.1283 \pm 0.0108,{ }t = 3.6682, corrected p = .0050$). There was no significant difference between DP and HC at the beta ($0.1336 \pm 0.0067{\text{ versus }}0.1378 \pm 0.0085,{ }t = 1.6990, corrected p = .0979$) and gamma ($0.1304 \pm 0.0081{\text{ versus }}0.1356 \pm 0.0076,{ }t = 2.0323, corrected p = .0743$) frequency bands in EC state. Consequently, it was the alpha band in EC state that was the condition with the best discrimination.

### Mean weighted directed brain network graph and its topological parameters

**Fig. 2:**
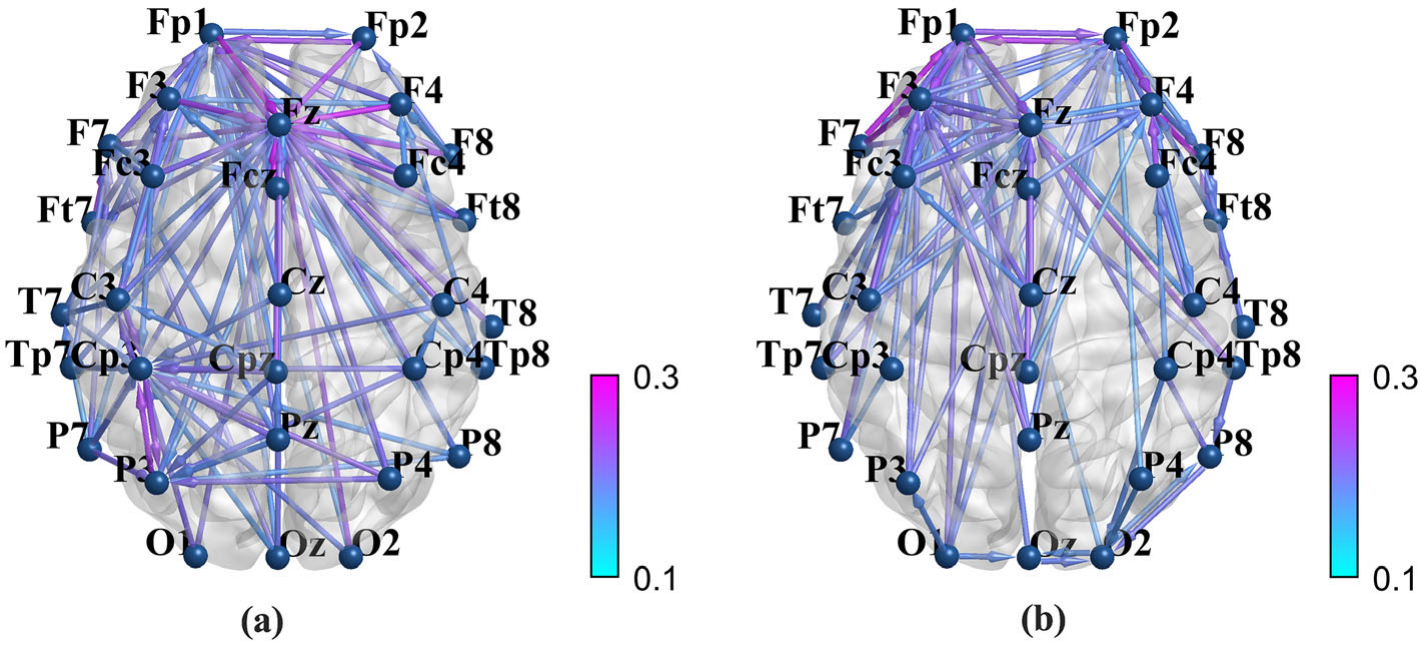
Mean weighted directed brain network graph of **a** healthy controls and **b** depressive patients. In order to facilitate observation, the connection weights between channels greater than 0.172 are retained

**Fig. 3:**
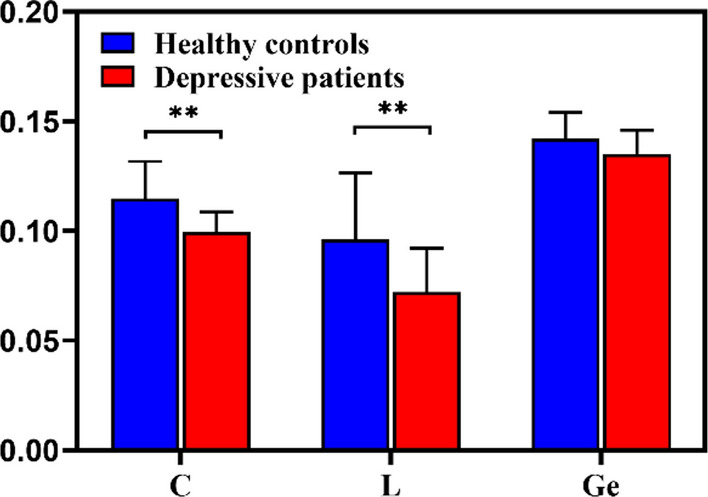
Topological parameters of directed functional networks in healthy controls (blue) and depressive patients (red). The mean and standard deviation are represented by bars and lines, respectively. The significant differences in the network properties between the two groups are denoted by double asterisks (corrected *p* < *.*01)

The mean PDC matrices were then converted to weighted directed brain network graphs at the alpha band in EC state. Mean brain network graphs for DP and HC are depicted in Fig. 2, in which the connectivity weights between channels greater than 0.172 are retained for easy observation. As can be seen, the brain disconnection phenomenon was observed in the depressive group. Then *C*, *G*_*e*_, and *L* were calculated to quantify the metrics of the graph, shown in Fig. 3. It is obvious that DP showed a significant (after correction) lower value for *C* and *L* ($C, 0.0997 \pm 0.0090\,{\text{versus }}\,0.1147 \pm 0.0169,{ }t = 3.4974, corrected \,p = .0023$;$L, 0.0722{ } \pm 0.0199{ }\,{\text{versus }}\,0.0960{ } \pm 0.0305{ },{ }\,t = 2.8989,\,corrected\,p = .0050$). This indicated a more random network structure in DP.

### Inter-hemispheric and intra-hemispheric functional connectivity

**Fig. 4:**
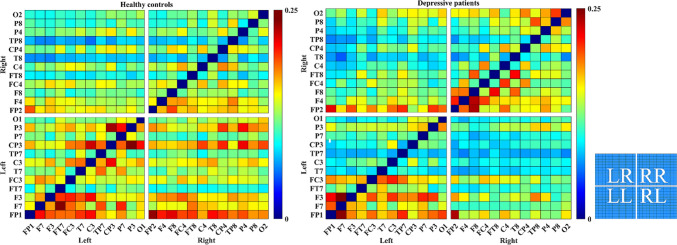
The mean weighted PDC matrices of different groups. Each matrix consists of four parts, representing the inter-hemispheric and the intra-hemispheric connectivity. The color bar is shown at the right of each matrix. The capital letters in the four quadrants of the small matrix mean left-to-left hemisphere (LL), left-to-right hemisphere (LR), right-to-right hemisphere (RR), and right-to-left hemisphere (RL), respectively

**Fig. 5:**
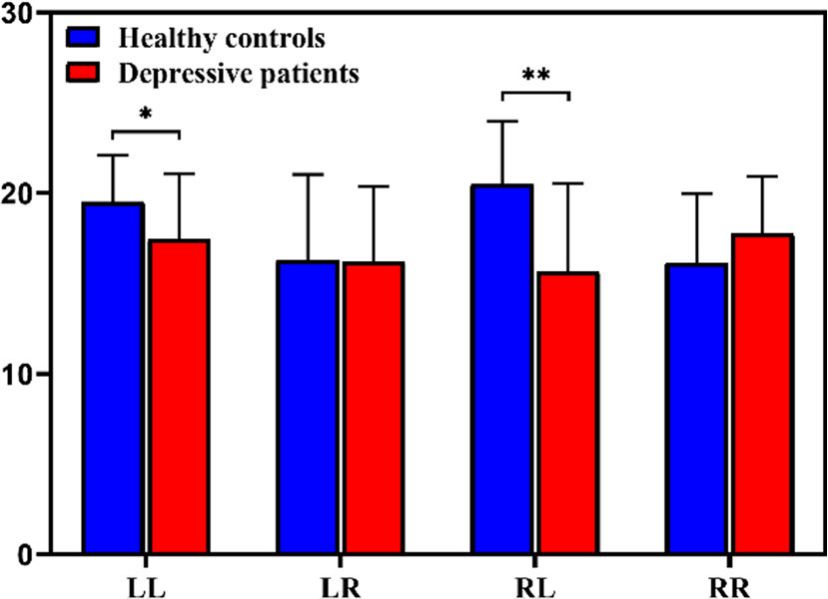
The inter-hemispheric (LR, RL) and intra-hemispheric (LL, RR) connectivity of healthy controls and depressive patients. The mean and standard deviation are represented by bars and lines, respectively, with blue for healthy controls and red for depressive patients. The asterisks denote corrected *p* < .05 (t-test) and double asterisks denote corrected *p* < .01 (t-test) after correction

To assess the alterations of information flow in depressive patients, we computed the mean inter-hemispheric and intra-hemispheric directed functional connectivity. 30 channels were divided into two parts, left hemisphere and right hemisphere. As depicted in Fig. 4, the matrix consists of four quadrants, of which the first quadrant indicates left-to-right (LR) hemisphere, the second indicates right-to-right (RR) hemisphere, the third indicates left-to-left (LL) hemisphere, and the fourth indicates right-to-right (RL) hemisphere. Obviously, the connectivity was disrupted in DP in both the inter-hemispheric (RL) and intra-hemispheric (LL) interactions. To evaluate quantitatively, the inter- and intra-hemispheric connectivity strength of each subject was then calculated and the statistical tests with BH-FDR correction were performed between two groups. As shown in Fig. 5, the DP had lower inter-hemispheric and intra-hemispheric connectivity compared with the HC, especially for LL ($17.44 \pm 3.62\,{\text{versus }}\,19.52 \pm 2.59,\,{ }t = 2.0540{ },\,p = .0481$) and RL ($15.64 \pm 4.91\,{\text{versus }}\,20.53 \pm 3.47,{ }\,t = 3.5744{ },\,p = .0023$) connectivity. This demonstrates that depression may interfere with the interactions inside the left hemisphere as well as from the right hemisphere to the left, which may be related with the left hemisphere asymmetry caused by the cortical deactivation of the right cerebral hemisphere reported in a prior study (Haag et al. 1994).

### Directed connectivity between different cerebral regions

**Fig. 6:**
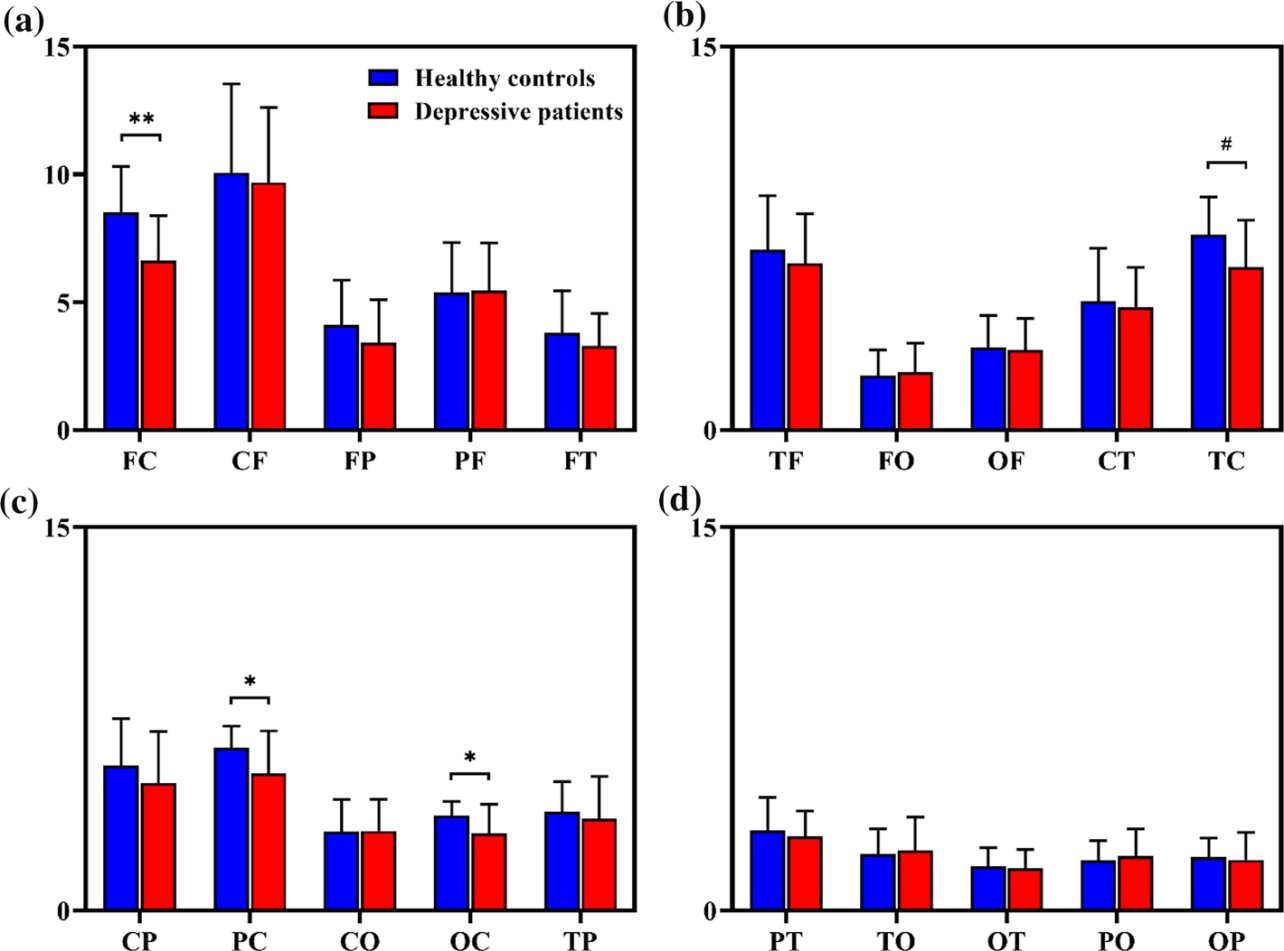
The directed connectivity of healthy controls and depressive patients between different regions. F, T, C, P, and O indicate frontal, temporal, central, parietal, and occipital, respectively. Combinations of different letters indicate directed connectivity between different regions (e.g. FC means frontal to central regions). The mean and standard deviation are represented by bars and lines, respectively, with blue for healthy controls and red for depressive patients. The asterisks denote corrected* p* < .05 (t-test) and double asterisks denote corrected *p* < .01 (t-test) and octothorpes denote corrected *p* < .06 (t-test)

Further, we also explored the connectivity strength between different cerebral regions. Thirty channels were divided into 5 regions: frontal (F, FP1, FP2, F7, F3, FZ, F4, F8), temporal (T, FT7, T7, TP7, FT8, T8, TP8), central (C, FC3, FCZ, FC4, C3, CZ, C4, CP3, CPZ, CP4), parietal (P, P7, P3, PZ, P4, P8), and occipital (O, O1, OZ, O2). The directed connectivity of each subject between different regions was calculated, and the significant differences were then tested with BH-FDR correction. The directed connectivity between different cerebral regions of DP and HC is shown in Fig. 6.

We found significant decreased connectivity in the frontal-to-central (FC,${ }6.6274{ } \pm 1.7606{ }\,{\text{versus }}\,8.5031{ } \pm 1.8066{ },\,t = 3.2836{ },\,{\text{corrected }}\,p = .0056{ }$), parietal-to-central (PC,${ }5.3759{ } \pm 1.6513\,{\text{versus}}\,{ }6.3801{ } \pm 0.8354{ },{ }\,t = 2.3776{ },\,{\text{corrected }}p = .0312{ }$), and occipital-to-central (OC,$2.9986{ } \pm 1.1553{\text{ versus }}3.7121{ } \pm 0.5514{ },{ }t = 2.4406{ },{\text{ corrected }}p = .0312{ }$) regions existed in DP. Besides, the connectivity strength in the temporal-to-central (TC, $6.3751{ } \pm 1.8356\,{\text{versus }}\,1.8356\, \pm \,1.4711{ },\,t = 2.3739{ },\,{\text{corrected}}\,p = .0583{ }$) regions also tended to show a lower value in DP. In general, the disrupted connectivity was mainly presented in the information flowing into the central parts, which may indicate that the central parts in depressive groups served as the local hubs appear to lose the function of integrating various types of sensory, cognitive and emotional information from other parts.

### Local topological parameters

To investigate the dysfunction of DP in local brain regions, the strength, out-strength, in-strength, and local efficiency of each subject were computed and compared between two groups. The significant differences after correction were highlighted by the bold words.

As presented in Table 1, both the strength and local efficiency of nodes located in central regions were lower in DP than HC. Furthermore, we found that the out-strength and local efficiency of frontal, occipital and parietal nodes were lower in DP. However, the significant difference of in-strength was only shown in C3 node. All these local findings indicate the abnormalities of functional segregation in DP, while functional segregation is important to specialized processing in brain (Mikail Rubinov and Sporns 2010).

**Table 1:** Local topological parameters including strenth, out-strength, in-strength, and local efficiency of different groups

	Healthy controls (n = 20)	Depressive patients (n = 19)	Corrected *p* value		Healthy controls (n = 20)	Depressive patients (n = 19)	Corrected *p* value
*The strength of different groups*							
FP1	9.08	8.38	0.4539	C4	7.53	6.67	0.1652
FP2	8.16	8.15	0.6051	T8	6.24	5.89	0.2664
F7	8.28	6.83	0.0944	TP7	7.23	6.06	0.1238
F3	8.36	8.23	0.5562	CP3	9.04	6.79	0.0944
FZ	9.68	8.31	0.1652	CPZ	7.89	7.01	0.1652
F4	7.88	8.01	0.6277	CP4	7.68	6.92	0.1761
F8	7.30	7.09	0.5146	TP8	6.26	6.16	0.5287
FT7	6.98	6.68	0.4611	P7	7.39	6.53	0.1761
FC3	7.82	7.88	0.6051	P3	8.59	7.56	0.2417
FCZ	7.57	7.07	0.2318	PZ	7.53	6.74	0.1652
FC4	7.76	7.06	0.1652	P4	7.57	6.73	0.1652
FT8	6.72	6.94	0.6818	P8	7.09	7.19	0.6277
T7	7.64	6.36	0.1238	O1	7.63	6.70	0.1238
C3**	**8.17**	**6.41**	**0.0022**	OZ	7.17	7.19	0.6051
CZ	7.52	6.51	0.1238	O2	7.16	7.67	0.7580
*The out-strength of different groups*							
FP1	3.64	3.27	0.0539	C4	3.82	3.51	0.0811
FP2*	**3.68**	**3.26**	**0.0314**	T8*	**4.03**	**3.68**	**0.0349**
F7	3.65	3.34	0.0811	TP7*	**4.00**	**3.72**	**0.0459**
F3	3.60	3.18	0.0557	CP3	3.83	3.84	0.5270
FZ	3.48	3.19	0.0869	CPZ*	**3.89**	**3.61**	**0.0459**
F4*	**3.75**	**3.29**	**0.0459**	CP4*	**3.90**	**3.52**	**0.0459**
F8**	**3.99**	**3.38**	**0.0051**	TP8*	**4.07**	**3.75**	**0.0314**
FT7*	**4.03**	**3.63**	**0.0314**	P7	3.99	3.70	0.0557
FC3	3.72	3.37	0.0516	P3	3.79	3.58	0.1881
FCZ	3.78	3.42	0.0811	PZ	3.99	3.72	0.0811
FC4*	**3.86**	**3.44**	**0.0459**	P4	3.91	3.60	0.0563
FT8*	**3.99**	**3.53**	**0.0280**	P8	3.97	3.70	0.0654
T7	3.92	3.81	0.2341	O1	3.88	3.71	0.1482
C3	3.71	3.62	0.3103	OZ*	**3.91**	**3.51**	**0.0314**
CZ*	**3.80**	**3.42**	**0.0459**	O2*	**3.89**	**3.55**	**0.0459**
*The in-strength of different groups*							
FP1	5.44	5.12	0.6735	C4	3.72	3.16	0.4675
FP2	4.48	4.88	0.8489	T8	2.21	2.21	0.7993
F7	4.63	3.49	0.3143	TP7	3.22	2.33	0.3605
F3	4.76	5.05	0.8489	CP3	5.21	2.94	0.2235
FZ	6.2	5.12	0.4675	CPZ	4.00	3.40	0.4675
F4	4.13	4.73	0.8598	CP4	3.78	3.40	0.5274
F8	3.30	3.72	0.8489	TP8	2.19	2.40	0.8489
FT7	2.95	3.05	0.8411	P7	3.40	2.83	0.4675
FC3	4.11	4.50	0.8489	P3	4.80	3.98	0.4800
FCZ	3.79	3.65	0.6735	PZ	3.54	3.01	0.4675
FC4	3.90	3.61	0.5679	P4	3.67	3.13	0.4675
FT8	2.73	3.41	0.8927	P8	3.12	3.49	0.8489
T7	3.72	2.55	0.3030	O1	3.75	2.99	0.3605
C3*	**4.46**	**2.79**	**0.0210**	OZ	3.26	3.69	0.8489
CZ	3.72	3.08	0.4675	O2	3.27	4.13	0.8780
*The local efficiency of different groups*							
FP1	0.1275	0.1129	0.0837	C4*	**0.1171**	**0.1013**	**0.0167**
FP2	0.1219	0.1128	0.1645	T8	0.1033	0.0948	0.1021
F7*	**0.1228**	**0.1033**	**0.0167**	TP7*	**0.1149**	**0.0958**	**0.0167**
F3	0.1248	0.1123	0.0587	CP3**	**0.1283**	**0.1044**	**0.0046**
FZ**	**0.1345**	**0.1136**	**0.0081**	CPZ*	**0.1229**	**0.1070**	**0.0239**
F4	0.1203	0.1137	0.1645	CP4*	**0.1203**	**0.1037**	**0.0167**
F8	0.1157	0.1056	0.1011	TP8	0.1040	0.0980	0.1645
FT7	0.1127	0.1011	0.0837	P7*	**0.1174**	**0.1003**	**0.0167**
FC3	0.1211	0.1114	0.1317	P3*	**0.1245**	**0.1102**	**0.0416**
FCZ*	**0.1168**	**0.1055**	**0.0254**	PZ*	**0.1182**	**0.1042**	**0.0254**
FC4*	**0.1199**	**0.1066**	**0.0278**	P4*	**0.1199**	**0.1033**	**0.0167**
FT8	0.1096	0.1041	0.2275	P8	0.1143	0.1072	0.1645
T7*	**0.1193**	**0.0997**	**0.0167**	O1*	**0.1194**	**0.1046**	**0.0199**
C3***	**0.1239**	**0.1011**	**0.0007**	OZ	0.1148	0.1070	0.1314
CZ*	**0.1172**	**0.1013**	**0.0167**	O2	0.1157	0.1093	0.1645

^*^Corrected *p* < 0.05

^**^Corrected *p* < 0.01

^***^Corrected *p* < 0.001

The significant differences after correction were highlighted by the bold words

### Correlation with HDRS scores

To explore the correlations between the network metrics and clinical symptoms of depressive patients, Pearson correlation analysis and linear regression were performed between the network metrics (*C, L*) and HDRS scores as well as the directed connectivity (LL, RL) and HDRS scores at alpha frequency band in the EC state. All of these metrics in depressive patients were significantly different from that in healthy controls. Correlation coefficients between HDRS scores and *LL* and *RL* were significant after correction ($LL:\mathrm{ r}=-0.5142,\mathrm{ corrected }p=0.0381, RL:\mathrm{ r}=-0.4787,\mathrm{ corrected }p=0.0381$). This finding indicate that the severity of depressive symptoms was negatively correlated with the intra- and inter-hemispheric connectivity.

## Discussion

In this study, we used partial directed coherence and graph theoretic analysis to investigate the differences of weighted directed brain network between DP and HC. We constructed the weighted directed brain network at alpha, beta, and gamma frequency bands in eyes-closed and eyes-open states. Topological characteristics and weighted directed functional connectivity were compared to find the hypofunction of brain network in DP. Our results indicated obvious alteration at the alpha band in the eyes-closed state.

Actually, abnormal alpha oscillations in depression have been repeatedly reported in many earlier studies (Fingelkurts et al. 2007; Gotlib et al. 1998; Zhang et al. 2018), but they have not yielded unified conclusions. We speculated that the inconsistent results may be caused by different brain patterns in different resting states including eyes-open and eyes-closed. Studies have also reported that brain in resting state maintains different patterns in eyes-open and eyes-closed condition, especially for alpha oscillations (Robert et al. 2007). Consequently, we designed the 8 min resting state experiment with eyes open and eyes closed in two alternating orders by voice playback, and analyzed eyes-open and eyes-closed states under different frequency bands. It was at the alpha frequency band in eyes-closed state that we found the most obvious alteration between DP and HC, thus we present our further results at alpha band in EC state only.

The results of topological characteristics showed decreased *C* and *L* in DP. Previous studies that used unweighted and undirected networks have reported lower *C* (Li et al. 2015; Sun et al. 2019; Zhang et al. 2018) and *L* (Hasanzadeh et al. 2020; Leistedt et al. 2009; Sun et al. 2019; Zhang et al. 2018) in patients with depression compared with healthy controls. It is stated that these alterations indicate a more random structure of depressive patients (Latora and Marchiori 2001), namely random network. Our observed more random network in depressive patients was consistent with these studies. This random structure in depressive patients is assumed to affect the cognitive capability of brain (Leistedt et al. 2009) and related to the abnormal changes of network hubs (Zhang et al. 2018). Random networks also show less modularized information processing capability or fault tolerance (Latora and Marchiori 2001; Zhang et al. 2011).

As mentioned above, to our knowledge, the weighted directed brain network has not been systematically tested in depression. The current study based on PDC is an attempt to expand the research on the weighted directed brain network in patients with depression. PDC is a promising tool that is better able to deal with multichannel data and identify the causality of interdependence between electrodes compared with conventional spectrum-based EEG analysis, thus providing more details of cortical functional interactions (Sun et al. 2008). Because the value of PDC can represent the direction between electrodes, we constructed the directed brain network, which was able to indicate the causality between brain regions. As the results showed in Fig. 5, the inter-hemispheric interactions (right-to-left) and the intra-hemispheric interactions (left-to-left) in depressed patients decreased significantly. Many earlier studies suggested that communication between the left and right cerebral hemispheres is a crucial component of cognitive and emotional processing (Banich et al. 1992; Compton et al. 2005; Toro et al. 2008). Inter-hemispheric communication of information is important for several reasons: to ensure that each hemisphere has access to crucial perceptual information about the world; and to allow for complex cognitive tasks to be allocated between the hemispheres in a manner that takes advantage of the cognitive specializations of each hemisphere (Banich et al. 1992). Thus, the result of decreased connectivity of the right-to-left hemisphere may cause an imbalance of information transmission between hemispheres, especially for right-to-left hemispheres. This abnormality in information processing of DP is consistent with a previous study that reported asymmetry to the left hemisphere, which interpreted as a cortical deactivation of the right cerebral hemisphere and seems to be a state marker of depression (Haag et al. 1994; Henriques et al. 1991). A study based on fMRI of depressed patients also found deficits in the interhemispheric connectivity in depressed patients (Wang et al. 2013).

The electrode activities of the midline were also considered. The connectivity strengths of the inner midline (MM), left hemisphere to midline (LM), midline to left hemisphere (ML), right hemisphere to midline (RM), and midline to right hemisphere (MR) were calculated. Only connectivity strength of ML was significantly (corrected, $p=0.02$) decreased in DP, which indicate the dysfunction of the left hemisphere in DP served as the information recipient.

As we can see in Fig. 6, weighted directed decreased connectivity of several specialized regions was presented. FC, PC, OC and TC connectivity was significantly lower in DP compared with HC. Our results also revealed that the outgoing information of the frontal, temporal, occipital and central parts in depressed patients were both decreased, but there was only decreased ingoing information in the left central parts (Table 1). These findings can be considered as a sign of lower information flow from frontal, temporal, occipital and parietal to left central regions in DP. The similar decreased results were also observed in local efficiency in DP, which means abnormality of functional segregation. We speculated that the central parts in depressive groups served as the local hubs appear to lose the function of integrating various types of sensory, cognitive and emotional information from other parts. In fact, cerebral activities have been investigated in the fronto-central and centro-parietal regions in patients with bipolar disorders (BD). A research about EEG alpha band suggest BD patients showed a decrease of mean synchronization in the alpha band, and the decreases were greatest in fronto-central and centro-parietal connections (Kim et al. 2013). Our findings support the reports about the cognitive decline and emotional disorders of depressed patients, which may provide new evidence about the disruptions of the interactions from frontal, temporal, occipital and parietal to central regions.

What’s more, to further highlight the advantages of the directed weighted brain network, we have constructed undirected brain networks using magnitude squared coherence (MSC) as a reference calculated by using HERMES, a toolbox on MATLAB (Niso et al. 2013). MSC measures the linear correlation between two variables $x\left( t \right)$ and $y\left( t \right)$ as a function of the frequency, $f$. We computed the same network metrics as we did earlier, and no significant results after correction were found. We found no statistical differences at any frequency band in EC or EO state $(p = 0.74, 0.23, 0.09, 0.46, 0.46\, and\, 0.24$ corresponding to alpha.EC, beta.EC, gamma.EC, alpha.EO, beta.EO and gamma.EO, respectively). Specifically, at alpha band in EC state, differences of the connectivity between the two hemispheres $\left( {p = 0.64} \right)$ and within the left hemisphere $\left( {p = 0.66} \right)$, as well as differences of the connectivity between different cerebral (frontal, temporal, occipital, parietal) regions and the central regions $(p > 0.05)$ and differences in topological characteristics ($p = 0.74, 0.70 \,and\, 0.74$ corresponding to *C*, *L* and *G*_*e*_, respectively), found in the directed network, did not appear in the undirected network. We speculated that undirected networks may confuse the direction of information flow in brain and lose the essential information in DP. Providing more useful information, directed brain networks can be considered as more effective tools for exploring the neurophysiological mechanisms of psychiatric disorders such as depression.

**Fig. 7:**
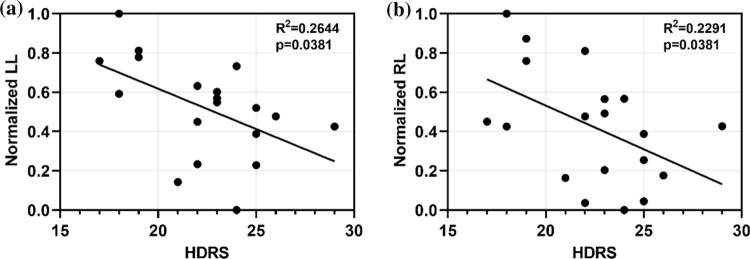
Correlation between HDRS scores and **a** normalized left to left hemispheric connectivity (*LL*), and **b** normalized right to left hemispheric connectivity (RL)

Figure 7 shows that the severity of DP is negatively correlated with two measured network metrics (*LL* and *RL*). DP with attenuated left to left and right to left hemispheric connectivity may have higher HDRS scores, indicating that the lower the *LL* and *RL*, the more severe the depression.

There are some limitations in our study. It must be acknowledged that the gamma rhythm is one of the popular approaches in studying neuromarkers of depression. Gamma rhythms are correlated with increased neuronal action potential generation (Nir et al. 2007; Watson et al. 2018). Many studies have indicated that gamma rhythms of HC are different from that of depressive patients (Akar et al. 2015; Lee et al. 2010; Liao et al. 2017; Liu et al. 2014; Pizzagalli et al. 2006; Siegle et al. 2010; Strelets et al. 2007). For example, an EEG study found that subjects with high depression scores (including Beck Depression Inventory (BDI) and Mood and Anxiety Symptom Questionnaire (MASQ) scores) had reduced resting gamma in the anterior cingulate cortex, whereas gamma increased in frontal and temporal regions in a study in which subjects with depression performed spatial and arithmetic tasks (Pizzagalli et al. 2006). In addition, subjects performing emotion-related tasks in major depression can show decreased frontal cortex gamma (Lee et al. 2010; Liu et al. 2014). Akar et al. found increased resting complexity of gamma signaling in the frontal and parietal cortex in subjects with major depression. All these studies prove that gamma rhythms are an important direction to explore the alterations of the brain in DP compared with heathy controls (Akar et al. 2015). However, we did not find significant differences at the gamma band. A possible reason is that we didn’t include cognitive tasks for DP. Our future study will further address the task-specific and sensory-based approaches for gamma rhythms. Besides, the number of subjects needed to be increased to make our results more convincing and we are working to recruit more subjects for further research.

In summary, we found decreased connectivity in DP at the alpha band during resting state with eyes closed. In addition, lower clustering coefficients and characteristic path lengths in DP indicate a more randomized network structure. Furthermore, the reduced inter-hemispheric (right-to-left) and intra-hemispheric (left-to-left) functional connectivity of DP suggest that DP have imbalance of information transmission coordination from right-to-left hemisphere, which may inhibit the expression of cognitive function. The decreased interaction from frontal to central, temporal to central, parietal to central and occipital to central in depressed patients suggest that the central parts in DP served as the local hubs appear to lose the function of integrating various types of sensory, cognitive and emotional information from other parts. Local findings including local efficiency and out-strength indicate the abnormalities of functional segregation in DP, while functional segregation is important to specialized processing in brain. What’s more, the directed network metrics may reflect an effective measure of the severity of depression. Based on our findings, we speculate that our research may serve as a potential neuromarker of the severity of depression.

## Data Availability

Data are available on request to the authors.
